# Do COVID-19 recovered individuals differ from healthy controls in accelerometer-measured physical activity and self-reported sleep? a UAE-based study

**DOI:** 10.1186/s12889-025-24779-6

**Published:** 2025-10-06

**Authors:** Ashokan Arumugam, Monica Raja Kumari Raghunathan, Mohammad Fawzi Safarini, Sangeetha Narasimhan, Sivapriya Ramakrishnan, Raneen Mohammed Qadah, Reime Jamal Shalash, Fatma Hegazy, Tamer Mohamed Shousha, George P. Nassis, Wael Daab, Sana Mehek, Yunus Nadeemulla Beig Khader, Nadia Rashed Al Mazrouei, Vennila J., Arto J. Pesola, Senthil D. Kumaran

**Affiliations:** 1 Department of Physiotherapy, College of Health Sciences, University of Sharjah, P.O. Box: 27272, Sharjah, United Arab Emirates; 2 Neuromusculoskeletal Rehabilitation Research Group, RIMHS – Research Institute of Medical and Health Sciences, University of Sharjah, P.O. Box: 27272, Sharjah, United Arab Emirates; 3 Sustainable Engineering Asset Management Research Group, RISE - Research Institute of Sciences and Engineering, University of Sharjah, P.O. Box: 27272, Sharjah, United Arab Emirates; 4 Department of Physiotherapy, Manipal College of Health Professions, Manipal Academy of Higher Education, Manipal, India; 5 College of Dental Medicine, University of Sharjah, Sharjah, United Arab Emirates; 6 Faculty of Physical Therapy, Cairo University, Cairo, Egypt; 7 College of Sport Science, University of Kalba, Kalba, Sharjah United Arab Emirates; 8 NMC Medical Centre, Shahba, Sharjah United Arab Emirates; 9 Amana Healthcare, Mubadala M42, Al Ain, United Arab Emirates; 10 College of Pharmacy, University of Sharjah, Sharjah, United Arab Emirates; 11 Manipal College of Health Professions, Manipal Academy of Higher Education, Manipal, India; 12 Preventive Health Research Unit, Active Life Lab, South-Eastern Finland University of Applied Sciences, Mikkeli, Finland; 13 Fibion Inc, Jyväskylä, Finland

**Keywords:** COVID-19, Sleep disorders, Physical activity, Accelerometer

## Abstract

The COVID-19 pandemic has significantly impacted global health, with recent studies suggesting long-term aftermath effects on physical activity (PA) and sleep among recovered individuals. Pre-existing challenges, such as high rates of physical inactivity and sleep deprivation in the United Arab Emirates (UAE), may exacerbate these effects. This cross-sectional study compared accelerometer-measured PA and self-reported sleep between 65 individuals who had recovered from COVID-19 within the past year (mean age 23.28 ± 6.05 years; 56 had acute COVID-19; 6 had ongoing symptoms up to 12 weeks, and 3 had post-COVID-19 syndrome) and 65 age- and sex-matched healthy controls (mean age 22.54 ± 4.32 years) in the UAE. Sociodemographic, anthropometric, and COVID-19 symptom history were collected through structured questionnaires. PA was measured using a thigh worn Fibion accelerometer for 4–7 consecutive days, including sitting, light, moderate, and vigorous intensity activity durations. Sleep was assessed using the Pittsburgh Sleep Quality Index (PSQI), Insomnia Severity Index (ISI), and Epworth Sleepiness Scale (ESS). Mann-Whitney U tests and chi-square analyses were used. No significant differences in sitting and PA durations were observed between the two groups. However, individuals recovered from COVID-19 exhibited longer sleep latency (29.15 min vs. 19.88 min, *p* = 0.033) compared to healthy controls. Other sleep parameters, such as sleep duration, efficiency, and daytime dysfunction, showed no statistically significant differences. Additionally, no associations were found between COVID-19 recovery and insomnia prevalence or excessive daytime sleepiness. These findings suggest that individuals primarily recovered from acute COVID-19 may not present with significant impairments in PA or sleep quality. However, the results also highlight the importance of individualized assessment and the need for further research to understand the broader spectrum of post-COVID cases (acute to post-COVID syndrome) and associated recovery outcomes.

## Introduction

Physical inactivity and sedentary behavior have been linked to a range of health issues, including cardiovascular diseases, diabetes mellitus, obesity, osteoporosis, and certain types of cancer [1, 2]. Moreover, different types of sedentary behavior, particularly prolonged television viewing, are consistently associated with various chronic diseases [3, 4]. Another study has also shown that even short periods of reduced physical activity, such as a two-week decrease in daily steps from 10,000 to 1,500, can impair insulin sensitivity, lipid metabolism, and cardiovascular fitness [5]. Adequate sleep is equally essential for maintaining metabolic health, cognitive function, and immune regulation. Disruptions in sleep patterns can further exacerbate the negative effects of physical inactivity, compounding risks to overall health.

The COVID-19 pandemic has had a significant impact on global health, with emerging evidence highlighting the long-term sequelae experienced by individuals recovering from the virus. One of the most common issues among people recovered from COVID-19 is fatigue. According to a research study by Kolokoltsev et al., people who recovered from COVID-19 had much less Physical Activity (PA) than they had before the infection [6]. Similarly, Katewongsa et al. explored the PA levels post-COVID-19 and found that many individuals struggle to return to their pre-pandemic activity levels [7].

Alongside fatigue, sleep disturbances have become increasingly common among individuals recovering from COVID-19. Studies have shown that individuals recovering from COVID-19 often experience insomnia, disrupted sleep patterns, and poor sleep quality [8, 9]. A recent case series reported lower sleep efficiency (based on the Pittsburgh Sleep Quality Index (PSQI)) in cases with severe respiratory symptoms and prolonged hospital stay as compared to others with mild respiratory symptoms due to COVID-19 [10]. Such sleep disturbances can have far-reaching consequences, given that restorative sleep is essential for both physical and mental health [11].

Both PA and sleep are key elements of health, and their importance is magnified in the context of post-COVID-19 recovery. Regular PA plays a vital role in promoting recovery, enhancing physiological resilience, and supporting long-term health outcomes, particularly important in the context of post-viral conditions such as COVID-19 [12]. However, persistent fatigue and breathlessness which is associated with long COVID can make it challenging for individuals to engage in regular PA, leading to a decline in overall fitness and functional capacity [13].

The UAE has been one of the countries most affected by the COVID-19 pandemic in the Middle East, with over 1 million confirmed cases and a recovery rate of approximately 98% as of October 2023 [14]. The UAE government implemented a series of stringent measures to curb the spread of the virus, including nationwide lockdowns, travel restrictions, and mass testing campaigns. These measures were largely successful in controlling the spread of the virus, but they also had unintended consequences, such as increased sedentary behavior and reduced PA levels among the population [15].

Even before the COVID-19 pandemic, the UAE faced significant challenges related to physical inactivity. The country has one of the highest rates of physical inactivity in the world, with only 19% of the population meeting the World Health Organization’s (WHO) recommended levels of PA [16]. This high prevalence of physical inactivity can be attributed to a combination of lifestyle factors, including extreme heat, urbanization, and cultural influences [17]. The UAE’s hot and dry weather makes it difficult to engage in outdoor PA almost the whole year [18]. Additionally, cultural factors, such as gender norms and societal expectations, have further contributed to low levels of PA, particularly among women [19].

In addition to physical inactivity, the UAE faces significant challenges related to sleep health. The country has one of the highest rates of sleep deprivation in the world, with approximately 40% of the population reporting insufficient sleep [15]. This high prevalence of sleep deprivation can be attributed to a combination of lifestyle and work-related factors. The UAE’s fast-paced, around-the-clock economy often requires individuals to work long hours or irregular shifts, which can disrupt sleep patterns.

Globally, most studies on post-COVID-19 PA and sleep have relied on self-reported measures, which are prone to bias and inaccuracies [12]. Self-reported data can be influenced by recall bias, social desirability bias, and subjective interpretations of PA and sleep quality. In contrast, objective measures, such as accelerometers and wearable devices, provide more accurate and reliable data on PA and sleep patterns [8, 18, 20].

A recent study examined the association of sociodemographic, anthropometric, and sleep quality factors with thigh-worn accelerometer-measured sitting and PA times among Emirati working women during the COVID-19 pandemic [19]. The findings revealed that Emirati working women spent an average of 11.6 h per day in sedentary activities. The study also found that poor sleep quality, as measured by the PSQI, was significantly associated with increased sedentary time and reduced PA levels. The use of accelerometers in this study provided a more accurate assessment of PA and sedentary behavior compared to self-reported measures, which are often prone to bias.

Another study by Arumugam et al. explored the association of sociodemographic factors with self-reported PA and sleep quality in Arab and non-Arab individuals during the COVID-19 pandemic [21]. They used the International Physical Activity Questionnaire Short Form (IPAQ-SF) and the PSQI to assess PA and sleep quality, respectively. The findings revealed significant differences in PA levels between Arab and non-Arab populations, with Arab individuals reporting lower levels of moderate to vigorous PA compared to the non-Arabs. The study also found that poor sleep quality was more prevalent among Arab participants, with 65% reporting suboptimal (poor) sleep (PSQI score > 5).

Despite global evidence, research in the UAE specifically comparing PA and sedentary behavior between post-COVID-19 individuals and healthy controls remains limited. Most studies in the UAE have focused on the general population’s PA and sleep patterns during the pandemic, without distinguishing between those who had COVID-19 and those who did not. The use of accelerometers in UAE studies represents a significant advancement in research on PA and sedentary behavior and provides objective, real-time data on PA and sedentary time [19, 22–24]. In contrast, studies relying on self-reported measures, such as the self-reported questionnaires, provide valuable but less precise data [21, 25]. Understanding the differences in PA, sedentary behavior and sleep between individuals recovered from COVID-19 and healthy controls would provide implications for developing targeted rehabilitation strategies and public health interventions in the UAE. This study aimed to compare accelerometer-measured PA levels and sleep parameters between individuals recovered from COVID-19 and age- and sex-matched healthy controls in the UAE.

## Methods

Participants were recruited using convenience sampling via social media, flyers, outpatient clinics hospitals, and word of mouth strategies. The participants’ recruitment strategy took place between September 2022 and April 2023. Written informed consent was obtained from all participants before enrolling them in the study. The study was reviewed and approved by the Research Ethics Committee, University of Sharjah (REC-22-03-10). All data were collected by three qualified research assistants who were physiotherapy graduates supervised by the research team.

Symptom history was collected based on the recommendations summarized in the Population-based age-stratified seroepidemiological investigation protocol for COVID-19 infection from the WHO (https://apps.who.int/iris/handle/10665/332188*)* and another COVID screening tool [26]. Symptom history mainly included assessment of fever, cough, chills, sore throat, shortness of breath (dyspnea)/breathing difficulties, nausea/vomiting, diarrhea, myalgia (body ache), general weakness, loss of appetite, loss of taste (ageusia), loss of sense of smell (anosmia), nosebleed, conjunctivitis, fatigue, seizures, irritability/confusion, altered consciousness, other neurological signs, and history of hospitalization. In addition, self-reported history of underlying conditions/comorbidities was recorded. All data were collected within a maximum of 12 months following COVID-19 infection. General sociodemographic information (age, sex, education, nationality, marital status, employment, etc.) was obtained via a self-reported questionnaire using Google forms. In addition, body weight was measured using a Tanita device and height was measured with a Seca portable stadiometer by two trained research assistants who were qualified physiotherapists. Measurements were taken without shoes and while participants wore light clothing. Body Mass Index (BMI) was calculated as weight in kilograms divided by height in meters squared (Kg/m^2^).

### Sitting and physical activity durations

A Fibion accelerometer (Fibion Inc, Jyväskylä, Finland) was distributed to participants on day one and collected on day eight. The device (20 g, L = 30 mm, W = 32 mm, T = 10 mm) was worn over skin of the right proximal thigh with hypoallergic Hypafix^®^ tape. The Fibion Device contains a tri-axial accelerometer, with embedded firmware that categorizes movement data into activity classes as light-intensity (< 3 METs), moderate-intensity (3–6 METs), and vigorous-intensity (> 6 METs), based on established criteria and computes energy expenditure [22, 27].

PA was monitored continuously 24 h a day over a period of 7 days. Participants were asked to always wear the device, including during sleep, and to remove it only during bathing, swimming or other water-based activities. The Fibion device measures raw acceleration in three axes and firmware-processed metrics (such as activity classifications and durations) can be exported for further analysis [28]. The following parameters were measured and used for analysis: time spent sitting as well as during light, moderate, and vigorous intensity activities. The device has been found to be reliable and valid to measure, identify and classify sedentary behavior and PA related metrics [29–32].

### Sleep parameters

The PSQI (Buysse, Reynolds III, Monk, Berman, & Kupfer, 1989; Carpenter & Andrykowski, 1998) was used to assess the (subjective) sleep quality (during the past month) of the individuals recovered from COVID-19. This is a self-report questionnaire with nine items for assessing seven components of sleep (quality, latency, duration, efficiency, disturbance, medication and dysfunction) with scores ranging from 0 to 21 (each component scoring ranges from 0 to 3). A lower score indicates better sleep quality. The PSQI has been reported to have good internal consistency, test-retest reliability, and construct validity (convergent/divergent validity and known-groups validity) when used for individuals with and without sleep problems [33].

The Insomnia Severity Index (ISI) is a brief, self-reported questionnaire designed to assess the nature, severity, and impact of insomnia. It consists of seven items that evaluate various aspects of insomnia, including difficulty falling asleep, staying asleep, waking up too early, satisfaction with current sleep patterns, interference with daily functioning, noticeability of sleep problems by others, and distress caused by sleep difficulties, all are rated based on the previous 2-week period. Each item is rated on a 5-point Likert scale, leading to a total score ranging from 0 to 28. Higher scores indicate more severe insomnia [34]. The ISI has demonstrated excellent internal consistency of 0.90 in a community sample and 0.91 in a clinical population, which indicates high reliability [34]. Also, the ISI has been shown to effectively differentiate between individuals with and without insomnia [35].

The Epworth Sleepiness Scale (ESS) is a self-administered questionnaire developed in 1991 to measure an individual’s general level of daytime sleepiness. It consists of eight items, each describing a common daily situation, such as sitting and reading or watching TV, the total score ranges from 0 to 24, with higher scores indicating greater daytime sleepiness. The ESS has demonstrated a high internal consistency of 0.88, for patients with a variety of sleep disorders [36]. A systematic review concluded that the ESS is a valid tool for assessing daytime sleepiness across various populations and settings [37]. All self-reported sleep related questionnaire data were collected using Google forms.

#### Data processing

Fibion data processing techniques reported in previous studies such as Pesola et al. and Yang et al. was employed in the current study to analyze sitting and PA [28, 32]. Fibion data was transferred to manufacturer’s website (www.fibion.com/upload) and in addition each participant’s age, gender, weight, and height were uploaded. As a result, explicit reports on sitting and PA times were obtained from the web service. Data in CSV files with minute-by-minute or day-by-day data was retrieved for subsequent analysis. Fibion data were normalized to a standardized 16-h daily wake time to account for variations in individual device wear time.The Fibion^®^ web service automatically analyses non-wear time as more than 30-minute periods when the device remains stationary. Any day with less than 10 h of wear-time was excluded from the analysis. Additionally, participants with fewer than 4 valid days of data (not including at least three weekdays and one weekend day) were omitted from the final analysis [38].

### Statistical analysis

Statistical analyses were performed using the IBM SPSS software, version 29 (IBM Corp., Armonk, NY, USA). Descriptive statistics of continuous data were presented as mean and standard deviation (SD), and categorical data were presented in the form of frequencies (with %). The Shapiro-Wilk test showed that the data did not meet the assumption of normality. Mann-Whitney U tests were used to compare sitting, PA and PSQI data between individuals recovered from COVID-19 and healthy controls. Effect size was calculated using a correlation coefficient (r) calculated as z/√N where z refers to z score and N refers to the total number of observations from both groups. Effect sizes for non-parametric tests were interpreted as follows: small (0.10–0.30), medium (0.30–0.50), and large (≥ 0.50). Chi-square analysis was used to assess the association of ISI and ESS scores with the COVID-19 status (those recovered from Covid-19 and healthy controls) and the results were presented as numbers and proportions (%). Statistical significance was set at *p* < 0.050.

## Results

One hundred and forty-five participants volunteered to participate in the study. However, eight were excluded due to technical issues with accelerometer data, and seven were excluded because their COVID-19 infection had occurred outside the inclusion time frame. As a result, a total of 130 participants was included in the study. Among them 65 had COVID19 (aged 23.28 ± 6.05 years) within the past one year and 65 were healthy controls (aged 22.54 ± 4.32 years). Table 1 summarizes demographic and anthropometric characteristics of participants. Table 2 presents the prevalence of various symptoms among individuals who have recovered from COVID-19 and healthy controls. Individuals who recovered from COVID-19 reported significantly higher rates of all listed symptoms compared to healthy controls, with fever, fatigue, headache, and cough being the most reported symptoms among recovered individuals.

**Table 1:** Demographic and anthropometric characteristics of participants (*n* = 130)

Variables	Individuals recovered from COVID-19 (*n* = 65)		Healthy controls (*n* = 65)	*p* value
Sex				
* Men*	13 (20.0%)		18 (27.7%)	0.303
* Women*	52 (80.0%)		47 (72.3%)	
Nationality				
* Gulf Cooperation Council (GCC)*	7 (10.8%)		8 (12.3%)	0.962
* Non-GCC Arab*	43 (66.1%)		42 (64.6%)	
* Non-Arab*	15 (23.1%)		15 (23.1%)	
Education				
* Bachelor’s degree*	53 (81.5%)		52 (80.0%)	0.621
* Master’s degree*	10 (14.4%)		12 (18.5%)	
* PhD*	2 (3.1%)		1 (1.5%)	
Marital status				
* Married*	9 (13.8%)		8 (12.3%)	0.693
* Not Married*	54 (83.1%)		53 (81.5%)	
* Prefer not to say*	2 (3.1%)		4 (6.2%)	
History of smoking				
* Yes*	4 (6.2%)		6 (9.2%)	0.510
* No*	61 (93.8%)		59 (90.8%)	
	Mean ± Standard deviation			
Age (years)	23.28 ± 6.05	22.54 ± 4.32		0.804
Height (cm)	165.46 ± 7.27	164.12 ± 9.13		0.162
Weight (kg)	67.86 ± 15.56	63.15 ± 12.83		0.096
Body mass index (kg/m^2^)	24.63 ± 4.60	23.35 ± 3.71		0.134

**Table 2:** Symptom history of participants during the COVID-19 pandemic (*n* = 130)

Symptoms		n (%)	Healthy controls (*n* = 65)
		Individuals recovered from COVID-19 (*n* = 65)	
Fever	*Yes*	55 (84.6%)	6 (9.2%)
	*No*	10 (15.4%)	59 (90.8%)
Sore throat	*Yes*	50 (76.9%)	5 (7.7%)
	*No*	15 (23.1%)	60 (92.3%)
Runny nose (rhinorrhea)	*Yes*	43 (66.2%)	3 (4.6%)
	*No*	22 (33.8%)	62 (95.4%)
cough	*Yes*	52 (80.0%)	4 (6.2%)
	*No*	13 (20.0%)	61 (93.8%)
Shortness of breath (dyspnea)	*Yes*	29 (44.6%)	4 (6.2%)
	*No*	36 (55.4%)	61 (93.8%)
Other respiratory symptoms	*Yes*	16 (24.6%)	1 (1.5%)
	*No*	49 (75.4%)	64 (98.5%)
Chills	*Yes*	36 (55.4%)	4 (6.2%)
	*No*	29 (44.6%)	61 (93.8%)
Vomiting	*Yes*	6 (9.2%)	1 (1.5%)
	*No*	59 (90.8%)	64 (98.5%)
Nausea	*Yes*	15 (23.1%)	2 (3.1%)
	*No*	50 (76.9%)	63 (96.9%)
Diarrhea	*Yes*	17 (26.2%)	0 (0.0%)
	*No*	48 (73.8%)	65 (100.0%)
Headache	*Yes*	52 (80.0%)	5 (7.7%)
	*No*	13 (20.0%)	60 (92.3%)
Muscle aches	*Yes*	46 (70.8%)	4 (6.2%)
	*No*	19 (29.2%)	61 (93.8%)
Joint ache	*Yes*	30 (46.2%)	3 (4.6%)
	*No*	35 (53.8%)	62 (95.4%)
Loss of appetite	*Yes*	27 (41.5%)	4 (6.2%)
	*No*	38 (58.5%)	61 (93.8%)
Loss of smell (anosmia)	*Yes*	30 (46.2%)	4 (6.2%)
	*No*	35 (53.8%)	61 (93.8%)
Loss of taste (ageusia)	*Yes*	29 (44.6%)	4 (6.2%)
	*No*	36 (55.4%)	61 (95.8%)
Fatigue	*Yes*	52 (80.0%)	4 (6.2%)
	*No*	13 (20.0%)	61 (95.8%)
Seizures	*Yes*	3 (4.6%)	0 (0.0%)
	*No*	62 (95.4%)	65 (100.0%)
Altered consciousness	*Yes*	10 (15.4%)	1 (1.5%)
	*No*	55 (84.6%)	64 (98.5%)
Other neurological signs (related to nerves)	*Yes*	6 (9.2%)	0 (0.0%)
	*No*	59 (90.8%)	65 (100.0%)
Hospitalization	*Yes*	6 (9.2%)	1 (1.5%)
	*No*	59 (90.8%)	64 (98.5%)

Fifty-six (86.2%) individuals recovered from COVID-19 experienced symptoms during the acute phase (up to 4 weeks) while 6 (9.2%) individuals had ongoing symptoms for up to 12 weeks. However, 3 (4.6%) individuals experienced post-COVID-19 syndrome (symptoms persisting more than 12 weeks). Sixty-one healthy controls without COVID-19 diagnosis were included. However, among other healthy controls, two had some clinical symptoms up to 4 weeks and two reported having had some symptoms more than four weeks but none of them were clinically diagnosed to have COVID-19.

## Sitting and physical activity times

Individuals who had recovered from COVID-19 had a median sitting time of 11.50 h per day, compared to 11.46 h in healthy controls. For light intensity activity, the median was 3.79 h in the COVID-19 recovered group and 3.65 h in the control group. Moderate intensity activity time was slightly higher in the healthy control group (48.43 min vs. 45.02 min). Similarly, the healthy control group had a higher median vigorous intensity activity time (1.32 min vs. 0.76 min). However, no statistically significant differences between the groups for sitting, and light, moderate, or vigorous intensity activity times were found (*p* > 0.050, Table 3).

**Table 3:** Differences in sitting and physical activity times (normalized to 16 h day) between individuals recovered from Covid-19 and healthy controls

Variable	Median (interquartile range)		z	Effect size	*P* value
	(Covid-19 recovered) (*n* = 65)	(Healthy controls) (*n* = 65)			
Sitting time (hr/16-h day)	11.50 (10.93 to 11.88)	11.46 (10.86 to 12.26)	0.217	0.02	0.829
Light intensity activity time (hr/16-h day)	3.79 (3.31 to 4.21)	3.65 (3.03 to 4.20)	−1.395	0.12	0.163
Moderate intensity activity time (min/16-h day)	45.02 (31.65 to 58.45)	48.43 (34.19 to 62.09)	1.273	0.11	0.203
Vigorous intensity activity time (min/16-h day)	0.76 (0.14 to 3.17)	1.32 (0.25 to 5.35)	1.669	0.15	0.095

### Sleep parameters

A statistically significant between-group difference was observed only for sleep latency (*p* = 0.03; Table 4), where individuals who had recovered COVID-19 took longer time to fall asleep (28.45 min) compared to healthy controls (19.88 min). There were slight differences in sleep duration, sleep efficiency, subjective sleep quality, sleep disturbances, use of sleep medication, and daytime dysfunction between the two groups; however, these differences were not statistically and clinically significant.

**Table 4:** Differences in Pittsburg sleep quality index (PSQI) scores between individuals recovered from Covid-19 and healthy controls

PSQI questionnaire parameters	Means ± SD		∆	Mann-Whitney U	Z	Effect size	*P* value
	Individuals recovered from Covid-19	Healthy controls					
Sleep latency (min)	29.15 ± 27.51	19.88 ± 18.70	−9.27	1660.50	−2.13	0.19	0.033
Sleep duration (h)	6.30 ± 1.70	6.27 ± 1.72	−0.03	2060.50	−0.25	0.02	0.807
Sleep efficiency (%)	90.97 ± 21.35	85.93 ± 18.43	−5.04	1956.50	−0.73	0.06	0.464
Subjective sleep quality (A.U)	0.95 ± 0.78	1.08 ± 0.835	0.13	2257.50	0.74	0.06	0.461
Sleep disturbances (A.U)	0.98 ± 0.63	0.92 ± 0.63	−0.06	2010.50	−0.58	0.05	0.562
Use of sleep medication (A.U)	0.31 ± 0.73	0.22 ± 0.60	−0.09	2011.00	−0.74	0.05	0.460
Daytime dysfunction (A.U)	1.00 ± 0.97	1.12 ± 1.01	0.12	2256.00	0.70	0.06	0.483

*AU* arbitrary units

There was no statistical association between COVID-19 status and insomnia prevalence. Similarly, the Epworth sleepiness scale revealed no significant association between COVID-19 and (excessive) daytime sleepiness (Table 5).

**Table 5:** Differences in insomnia severity index and Epworth scale scores between individuals recovered from Covid-19 and healthy controls

Variables	*n* (%)		Chi-Square value	*p* value
	Individuals recovered from Covid-19	Healthy controls		
Insomnia severity index				
*Have insomnia*	8 (12.3%)	16 (24.6%)	3.27	0.070
*Doesn’t have insomnia*	57 (87.7%)	49 (75.4%)		
**Epworth scale**				
*Unlikely to be abnormally sleepy*	46 (70.8%)	52 (38%)	1.49	0.220
*Excessively sleepy*	19 (29.2%)	13 (9.5%)		

## Discussion

The current study aimed to evaluate differences in PA levels and sleep parameters between individuals who had recovered from COVID-19 and age- and sex-matched healthy controls in the UAE. No statistically significant differences were observed between the two groups for accelerometer-measured sitting and PA times and most self-reported sleep parameters.

PA of individuals was drastically reduced globally during the COVID − 19 pandemic [39]. A recent study reported that post-hospitalized patients with COVID-19 experienced high levels of sedentary time 3–6 months after hospitalization, while PA and sedentary behavior were not impacted by patient or disease characteristics [40]. In our sample, only six individuals reported being hospitalized due to their COVID-19 symptoms. PA levels, particularly moderate to vigorous intensity activity, have been shown to decrease during the COVID-19 pandemic. This trend has been reported in both infected and non-infected individuals, although specific comparisons between individuals recovered from COVID-19 and healthy controls are not detailed [41].

In our study, all data were collected from COVID-19 group within 12 months of infection, without categorizing them based on illness severity (e.g., mild, moderate, or severe disease). Among our participants in the COVID-19 group, 64 participants (88%) suffered from acute COVID-19 (symptoms duration < 4 weeks), which is the milder form of the disease thereby minimizing the impact on long-term PA and sleep. Furthermore, less severe COVID-19 infections may not have caused lasting physiological or psychological disturbances that could significantly alter PA or sleep behavior. A systematic review revealed that individuals with more severe illness or those experiencing long COVID symptoms may exhibit greater disruptions in physical functioning and sleep quality [41]. However, variations in study populations, the severity of illness, time since recovery from COVID- illness, and the assessment tools may explain the discrepancies noted among our current study and previous investigations.

Our study results did not demonstrate any significant difference in most sleep parameters between groups. Among all the sleep parameters studied, significant between-group differences are observed only for sleep latency (*p* > 0.031) as assessed by the PSQI. This finding is in agreement with that of Carnes-Vendrell et al. who demonstrated higher sleep latency in individuals affected by COVID-19 compared to healthy controls [42]. They also reported poorer sleep between groups [42]. Individuals who have recovered from COVID-19 may experience more sleep disturbances compared to those who have never been infected. This includes poor subjective sleep quality, prolonged sleep latency, shorter sleep duration, reduced sleep efficiency, and frequent daytime dysfunction [43]. Another study found that health professionals infected with COVID-19 have poorer sleep quality, associated with excessive daytime sleepiness [44]. There could be several other factors that might have accounted for the lack of significant differences in PA and sleep patterns between the groups in our study. First, it is plausible that participants had ample time to regain their pre-COVID activity levels and sleep quality, especially as the infection occurred within 12 months prior to data collection. This recovery period may have allowed individuals to re-establish their regular lifestyle routines. Additionally, individuals recovering from COVID-19 might have become more health-conscious and deliberately adopted healthier habits, including improved sleep hygiene and more regular PA. These proactive behavior changes could have contributed to PA and sleep parameters comparable to those of the healthy control group.

Another explanation could lie in the measurement tools used. The use of accelerometers provided objective data on PA and sedentary behavior, reducing reliance on subjective reporting [21, 24]. Additionally, the use of a validated sleep questionnaire allowed for standardized assessment of sleep quality across participants. Alternatively, while accelerometers offer objective and reliable measures of movement, they may not capture all dimensions of PA, such as perceived exertion, upper body movements, or load-bearing tasks [29, 45, 46]. Likewise, sleep questionnaires such as the PSQI and other questionnaires used in our study are only self-reported measures of sleep quality and may not fully capture subtle sleep disturbances or physiological changes in sleep architecture [47].

## Methodological considerations, limitations and future recommendations

We did a post hoc power analysis for sitting and PA and sleep variables. The analysis revealed high statistical power (≥ 0.85) for detecting small effect sizes in light, moderate, and vigorous intensity PA, as well as sleep latency and sleep efficiency, implying adequate sensitivity to identify between-group differences. Even so, the statistical power was low for sitting time and sleep duration (power ≈ 0.22), reflecting the very small effect sizes observed and suggesting a higher risk of Type II error for these outcomes. Despite the statistical significance, the observed between-group difference in sleep latency (≈ 9 min) may be of limited clinical significance. Nevertheless, the consistently small effect sizes across all variables (0.02–0.15) suggest the absence of clinically meaningful between-group differences in PA and sleep parameters.

Both recovered COVID-19 individuals and healthy controls exhibited high levels of daily sedentary time, representing a significant concern within this population sample regardless of COVID history. The 2020 WHO Guidelines emphasized that adults and older adults should limit sedentary time and replace prolonged sitting with PA. They recommended breaking up long periods of sitting with PA of any intensity and meeting the minimum PA targets (150–300 min of moderate or 75–150 min of vigorous activity weekly). The guidelines highlighted that even small reductions in sedentary behavior can improve health outcomes, reducing risks of cardiovascular disease, diabetes, and premature mortality [48].

The self-reported nature of the sleep assessments could introduce reporting bias or inaccuracies. Moreover, the sample may not be representative of the broader population, as the majority of our participants (86%) did not experience post-COVID-19 syndrome or long COVID symptoms, and the convenience sampling method could have introduced selection bias. Clinical indicators of COVID-19 severity other than the duration of symptoms (e.g., hospitalization duration, use of oxygen therapy, intensive care unit admission) were not collected, which precludes exploring severity-specific differences and may restrict the generalizability of findings to individuals with more severe illness.

The mean BMI was slightly higher in the COVID-19 recovered group compared to healthy controls, but this difference was not statistically significant. However, the influence of BMI on PA and sleep outcomes warrants consideration in future studies, specifically those comparing individuals across different BMI categories. Psychosocial factors were not examined in this study and should be incorporated in future research to provide a more holistic perspective on the factors influencing PA and sleep.

Future research should aim to address these limitations by including a more diverse sample of participants representing varying degrees of COVID-19 severity, recovery timelines, and different BMI categories. Incorporating a combination of polysomnography, heart rate monitors, accelerometry, biomarkers, and self-reported measures could provide deeper insights into the true impact of COVID-19 on physical and sleep health. The cross-sectional design of the present study does not allow for assessment of individual trajectories or comparison with pre-infection baseline data, which were not available. Therefore, causal inferences between COVID-19 and PA or sleep parameters cannot be drawn. Prospective longitudinal cohort studies are also warranted to track recovery trajectories over time, while mixed method studies could provide further insights into individual experiences and adaptations during the post-recovery phase.

## Conclusion

This study found no significant differences in PA levels and nearly all sleep parameters between individuals recovered from COVID-19 and age- and sex-matched healthy controls. These findings primarily suggest that, for most individuals who recovered from acute COVID-19, the infection may not lead to long term impairments in PA or sleep. However, both groups exhibited high sitting time and low vigorous intensity activity levels, highlighting the need for reducing sedentary behavior and promoting PA as required. There is a need for individualized assessment, and further research exploring post-COVID recovery outcomes depending on the severity of the infection in the long term is required.

## Data Availability

Data are available from AA upon reasonable request.
